# EEPD1 regulates inflammation and endothelial apoptosis in atherosclerosis through KLF4‐EEPD1‐ERK axis

**DOI:** 10.1002/ctm2.70311

**Published:** 2025-04-23

**Authors:** Kaiwen Yu, Xiang Li, Xin Shi, Ruogu Li, Min Zhang

**Affiliations:** ^1^ Department of Cardiology Shanghai Jiao Tong University Affiliated Chest Hospital Shanghai China

**Keywords:** apoptosis, atherosclerosis, EEPD1, endothelia

## Abstract

**Background:**

Inflammation and endothelial apoptosis are implicated in the advancement of atherosclerosis. EEPD1 holds a pivotal position in the repair of DNA damage and contributes to the progression of multiple cancers. However, the role of *EEPD1* in cardiovascular diseases needs to be explored further, especially in atherosclerosis.

**Methods:**

We constructed *EEPD1* and ApoE (apolipoprotein E)‐deficient mice to assess how EEPD1 influences endothelial inflammation and apoptosis within atherosclerotic plaques. High‐throughput RNA sequencing of human aortic endothelial cell groups treated with siCon+TNFα and si*EEPD1*+TNFα identified notable disparities in the MAPK pathway between groups. Chromatin immunoprecipitation and luciferase reporter assay confirmed that KLF4 directly regulates EEPD1.

**Results:**

Further examination of gene expression data revealed elevated EEPD1 concentrations in atherosclerotic plaques of patients, which findings were corroborated in the aortas of ApoE^−/−^ mice. Present study demonstrated that adhesion molecule expression, endothelial apoptosis, aortic root plaques and macrophage accumulation were markedly ameliorated in *EEPD1* ^−/−^ *ApoE* ^−/−^ mice compared to WT *ApoE* ^−/−^ mice. Functional analysis revealed that increase in *EEPD1* promotes *ERK* phosphorylation and significantly increases endothelial apoptosis and inflammation in atherosclerosis, which was abrogated by inhibition of *ERK* phosphorylation. We found *KLF4* to be the transcription repressor of *EEPD1* through luciferase assay and chromatin immunoprecipitation, and *KLF4* inhibition abrogated the amelioration of endothelial apoptosis and inflammation caused by *EEPD1* deletion.

**Conclusions:**

Collectively, this study revealed that EEPD1 deletion can lead to amelioration of atherosclerosis through the KLF4‐EEPD1‐ERK axis. Hence, targeting EEPD1 could be a promising therapeutic strategy for patients with atherosclerosis.

## Graphical abstract

Inflammation reduces transcriptional inhibition on EEPD1 by lowering KLF4 levels. Elevated EEPD1 subsequently activates ERK phosphorylation. This process exacerbates endothelial inflammation and apoptosis, thus accelerating atherosclerosis development

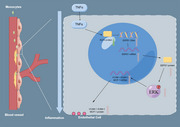

## 1 INTRODUCTION

Atherosclerosis is the major cause of cardiovascular diseases and a leading contributor to global morbidity and mortality. The integrity of the endothelium is indispensable for vascular homeostasis as it functions as both a barrier and a responder to circulating signals.^1^, ^2^ Adhesion molecules and chemokines, such as vascular cell adhesion molecule‐1 (VCAM‐1), intercellular cell adhesion molecule‐1 (ICAM‐1), and monocyte chemotactic protein‐1 (MCP‐1), facilitate the recruitment and attachment of monocytes to the endothelial surface, and infiltration into the vascular wall at arterial curvatures and bifurcations wherein disturbed flow (DF) activates the endothelium.^3^ These processes play a pivotal role in the onset and progression of atherosclerosis.

Endonuclease/exonuclease/phosphatase family domain‐containing 1 (*EEPD1*) experiences an upregulation in embryonic stem cells after DNA damage. It initiates end resection and promotes recombination via homology against classical nonhomologous end‐joining and end joining facilitated by microhomology. Several studies have suggested a vital function of *EEPD1* in the processes of multiple diseases, including esophageal squamous cell carcinoma,^4^ acute myeloid leukaemia,^5^ and breast cancers.^6^ Nonetheless, its function in cardiovascular disease remains unclear, particularly with regard to atherosclerosis.

Kruppel‐like factor 4 (*KLF4*) is a transcription factor within KLF family that functions as a regulator in proliferation, apoptosis, and somatic cell reprogramming. Human *KLF4* is localised on chromosome 9q31.^7^ The molecules are cloned, and the corresponding protein comprises 513 amino acids. This protein contains domains for transcriptional activation and suppression, which bind to a responsive element containing the CCACC core sequence.^8^, ^9^ *KLF4* also exerts a crucial influence on many cardiovascular disorders, namely, atherosclerosis,^10^ cardiac hypertrophy,^11^ nonischemic cardiomyopathy,^12^ and myocardial ischemia‐reperfusion.^13^ However, the association between *EEPD1* and *KLF4* has not yet been elucidated.

Gene expression within the MAPK pathway is implicated in various cellular processes, including proliferation,^14^ motility,^15^ adhesion,^16^ apoptosis,^17^ and glucose metabolism.^18^ After being activated, ERK moves to the nucleus and phosphorylates various substrates, including transcription factors like CREB and Elk1. The stimulation and inhibition of nuclear targets promote growth and proliferation while inhibiting cell death. Furthermore, *ERK* plays a significant role in several cardiovascular diseases like cardiac ischemia reperfusion,^19^ cardiac hypertrophy,^20^ and diabetic cardiomyopathy.^21^ Nevertheless, the specific regulatory interactions between EEPD1 and ERK in the context of atherosclerosis have not been clarified.

In our research, we revealed that *EEPD1* is elevated in atherosclerosis and enhances *ERK* phosphorylation while deteriorating endothelial function and apoptosis; these impacts are further intensified by *KLF4* suppression. In summary, KLF4‐EEPD1‐ERK axis plays a pivotal function in the pathogenesis of atherosclerosis.

## 2 METHODS

### 2.1 Animal study

Using the CRISPR‐Cas9 system, the Shanghai Nanfang Research Center for Model Organisms developed an EEPD1 knockout (EKO) mice. During the process, four guide RNAs sliced through the EEPD1 genome sequence. By mating EEPD1^–/–^ mice with ApoE^–/–^ mice, EEPD1^–/–^ApoE^–/–^ double knockout mice were bred. The Shanghai Jiao Tong University Animal Care Committee reviewed and granted approval for all procedures involving animals. All experiments and measurements were performed under blinded conditions. Eight‐week‐old adult males were chosen as experimental subjects. The research focused exclusively on male mice, considering estrogen's known impact on atherosclerosis development.^22^, ^23^ All animals were maintained in an environment with a 12‐h light/dark cycle, housed at 23°C and kept within humidity ranging from 40% to 70%. They were fed a high‐fat diet (HFD, containing 1.25% cholesterol and 0.5% sodium cholate, specifically D12109C from Research Diets, New Brunswick, NJ, USA) for a duration of 12 weeks. Eight‐week‐old ApoE^–/–^ mice were administered a 200 µL injection of *EEPD1* overexpression vector (pAAV‐hFLT1‐*EEPD1*‐3×Flag‐P2A‐mNeonGreen‐tWPA) or empty vector (pAAV‐hFLT1‐3×Flag‐P2A‐mNeonGreen‐tWPA), containing  a suspension with 1×10^12^ copies, through tail vein. An intraperitoneal administration of pamoic acid (100 mg/kg every alternate day; No. HY W008613, MedChemExpress) and PBS was administered during the last 4 weeks in mice. After 12 weeks of high‐fat feeding, the mice were subjected to tissue harvesting. Prior to this procedure, mice were anaesthetised using 30 mg/kg body weight 1% pentobarbital sodium in saline through intraperitoneal injection to minimise their suffering and distress. In our study, rigorous and humane procedures were implemented for verifying the death of mice to ensure the accuracy of experimental data and the respect for animal welfare. The specific steps taken are as follows: Initially, we assessed the vital signs of the mice, including respiration, heart rate, and reflexes, to determine if they were deceased. Mice under deep anaesthesia or in a state of natural death cease respiration, their heart stops beating, and they exhibit no response to pain stimuli such as a toe pinch. These observations served as the primary basis for our initial determination of death. Following the initial judgment of death, we performed a secondary confirmation to ensure accuracy. This typically involved rechecking the mouse's respiration and heart rate, as well as employing more sensitive methods (e.g., stethoscope or electrocardiogram) to verify the cessation of vital signs. In order to ensure the welfare of experimental animals and adhere to ethical principles, we established well‐defined humane endpoints. Throughout the experimental process, we routinely monitored key indicators including animal weight, food intake, activity levels, and behavioural patterns. When animals exhibited overt signs of distress indicative of pain, such as persistent vocalisations, self‐mutilation, or abnormal posturing, they were considered to have reached the humane endpoint criteria.

### 2.2 Oil Red O staining

Oil Red O staining was used to quantify the atherosclerotic plaque lesion area. During en face analysis, the abdominal aortic arteries were collected and cut open lengthwise with the intima facing outward. They were then rinsed with tap water for 5 s before being stained with Oil Red O reagent (G1016, Servicebio) at 37°C for an hour. Afterward, arteries were extracted and exposed to 75% isopropanol. The resulting differentiation was halted once the fatty plaques in the lumen turned orange or bright red; then the images were captured under a Stereomicroscope. The aortic sinus cross‐sections underwent fixation, dehydration and staining with Oil Red O.

### 2.3 Cell culture and treatment

Primary human aortic endothelial cells (HAECs) were acquired from American Type Culture Collection (Catalogue No. PCS‐100‐011, Lot no. 63233442; Manassas, VA, USA) and grown in DMEM (Gibco, USA) enriched with 10% fetal bovine serum (Gibco). Tumour necrosis factor‐alpha (TNFα) was obtained from Novoprotein (DC008). Prior to assay, HAECs were exposed to 20 ng/mL of TNFα for 24 h.^24^

### 2.4 Small‐interfering RNA (siRNA) transfection

HAECs were grown in six‐well dishes until 70%–80% confluency and transiently transfected with 3 µg *EEPD1* siRNA (Tsingke, Beijing, China) or scrambled siRNA (Tsingke) for 48 h. Then, HAECs were were exposed to  20 ng/mL TNFα for a period of 24 h. Below are the sequences of the siRNAs: si‐*EEPD1*, 5′‐CGAAGUCUCUGGACAACAU‐3′, si‐*KLF4*, 5′‐GCAGCUUCACCUAUCCGAU‐3′, si‐NC 5′‐UUCUCCGAACGUGUCACGU‐3′.

### 2.5 Immunofluorescence

After permeabilisation, sections were incubated with 5% bovine serum albumin for 1 h, and subsequently incubated with goat anti‐platelet endothelial cell adhesion molecule (CD31) (1:200, AF3628‐SP, R&D), rabbit anti‐*EEPD1* antibody (1:200, HPA053668, Sigma), rabbit anti‐ICAM‐1 antibody (1:200, ab109361, Abcam), and mouse anti‐*KLF4* (1:200, sc‐393462, Santa Cruz) antibodies at 4°C overnight. Following three rinses with PBS, the sections were maintained in the presence of secondary antibodies at ambient temperature for an hour. Following three rinses with PBS, tissues were immunostained with antifade mounting medium with DAPI (P0131, Beyotime Biotechnology) and observed under a microscope manufactured by Leica.

### 2.6 Real‐time polymerase chain reaction

Real‐time polymerase chain reaction was performed on an Applied Biosystem 7300 plus Sequence Detection System (Applied Biosystems) using an SYBR Green Polymerase Chain Reaction kit (Q511‐02, Vazyme). Using 2^−ΔΔCt^ method, normalisation of the target gene expression was achieved. Below are the sequences of the primers (5′ to 3′): KLF4 f: CCGCTCCATTACCAAGGTCAGTC, KLF4 r: ACGGTAGTGCCTGGTCAGTTCA, EEPD1 f: CTGGAAGGCTGTTGTTGCTGAGAA, EEPD1 r: CTTGGTGCTGATGTTGGTGAAGGT.

### 2.7 Gene set enrichment analysis

GSE100927 and GSE43292 were obtained from the Gene Expression Omnibus (GEO) database. R language was employed for examining the difference of EEPD1 between atherosclerosis and control groups in GSE100927. We conducted analysis using R language to investigate the differential expression of EEPD1 between atherosclerotic group and control group in GSE100927, as well as the differential expression of EEPD1 between advanced stage plaques and early stage plaques in GSE43292.

### 2.8 Western blot

An equivalent of protein was separated by sodium dodecyl sulphate‐polyacrylamide gel electrophoresis (SDS‐PAGE) and transferred to polyvinylidene difluoride membranes (Millipore). Subsequently, membrane was blocked for 1 h and probed with the primary antibodies against *EEPD1* (1:1000, HPA053668, Sigma), VCAM‐1 (1:1000, ab184247, Abcam), ICAM‐1 (1:1000, sc‐8439, Santa‐Cruz), MCP‐1 (1:1000, ab214819, Abcam), *KLF4* (1:1000, A13673, ABclonal), Bcl2 [1:1000, 3498, Cell Signaling Technology (CST)], Bax (1:1000, 14796, CST), phospho‐*ERK* (1:1000, 4370, CST), total‐*ERK* (1:1000, 4695, CST), and GAPDH (1:1000, 5174, CST). After rinsing, membranes underwent incubation with the secondary antibody for 60 min at ambient temperature. Chemiluminescence was detected utilising an Amersham Imager 680 (Amersham).

### 2.9 Apoptosis assay

Apoptosis was assessed with a TUNEL assay kit (T2130, Solarbio). Briefly, the experimental procedure was carried out as follows. We rehydrated and fixed sections for 2 h in Bouin's Solution (HT10132, Sigma). The section was incubated at 37°C for 30–60 min after 50 µL of TUNEL working solution was added to each sample. Then we washed sections with PBS. After adding 100 µL Reaction buffer (Component B) to each sample, we incubate sections for 20 to 30 min. The nuclei were stained with DAPI (G1012, Servicebio). Finally, the sections were observed under fluorescence confocal microscope (Carl Zeiss, Germany) with a 488 nm excitation filter.

### 2.10 Masson's staining

We rehydrated and fixed sections for 2 h in Bouin's Solution (HT10132, Sigma). Next, the Masson Ponceau Magenta staining solution was applied dropwise for 5 min. After rinsing the sections with the diluted acid solution, a 1% aqueous solution of phosphomolybdic acid was added in droplets to differentiate the tissues for a duration of 3 min, followed by immersion in aniline blue solution for 1 min of staining. Ultimately, the samples underwent dehydration through a series of ethanol concentrations, were rendered transparent via xylene treatment, and were then encapsulated with neutral resin.

### 2.11 Flow cytometry

Annexin‐FITC apoptosis detection kit (Beyotime) was used to assess the levels of apoptosis. HAECs were seeded in 6‐well plates and exposed to TNFα (20 ng/mL) or control (Ctrl) culture media for 24 h. Subsequently, HAECs were collected using pancreatin (EDTA‐free) and rinsed twice with chilled PBS. Afterward, cells were incubated in the dark for 20 min with Annexin‐FITC reagent. The apoptosis rate was measured on the C6 Flow Cytometer™ system (BD Biosciences, CA, USA).

### 2.12 Monocyte adhesion assay

HAECs were exposed to 20 ng/mL TNFα or vehicle for 24 h with or without 20 µmol/L 1 mmol/L pamoic acid incubation or KLF4 siRNA transfection. The human THP‐1 monocytes, at a concentration of 1×10^6^ cells per millilitre, were re‐dispersed in RPMI 1640 medium devoid of serum and labelled with 2 µmol/L calcein‐AM (No. C2012, Beyotime) for 30 min. HAECs were cultured in DMEM without FBS and incubated with human THP‐1 monocytes at 37°C for 1 h. Finally, cells underwent three rinses using PBS to eliminate the nonadherent human THP‐1 monocytes and fixed with 4% paraformaldehyde. The attachment of human THP‐1 monocytes to HAECs was examined using a Leica microscope.

### 2.13 Luciferase activity assay

The promoter region sequence of EEPD1 was retrieved from NCBI. (https://www.ncbi.nlm.nih.gov/). JASPAR (http://jaspar.genereg.net/) was used to examine potential KLF4 protein binding sites in the EEPD1 promoter. The promoter sequence of *EEPD1* containing *KLF4* binding sequence was subcloned into a pGL3 promoter luciferase vector; the insert sequence was validated by DNA sequencing. HAECs were plated in a 24‐well dish and simultaneously transfected with 0.25 µg of each plasmid along with 25 µg of phRL‐TK (Promega), which harbours the Renilla luciferase gene as an internal control for transfection efficiency, employing Lipofectamine 3000. The activity of luciferase was assessed employing the Promega Dual‐Luciferase Reporter system.

### 2.14 Chromatin immunoprecipitation (ChIP) assay

ChIP assays followed previous methods.^25^, ^26^ The chromatin DNA was prepared from HAECs using a commercial kit (P2083S, Beyotime). HAECs underwent transfection of KLF4 siRNA, subsequently cross‐linked in 1% formaldehyde. The previously characterised *KLF4* polyclonal or control rabbit IgG was used for the immunoprecipitation of the protein‐DNA complexes from chromatin.

### 2.15 Transcriptome and data analysis

The transcriptome was analysed using a HiSeq X Ten instrument (OE Biotech Co., Ltd, Shanghai, China). The HISAT2 program was used to map the sequenced reads to the mouse genome.^27^ Using htseq count, the read counts were determined and then aligned to the gene's exonic regions according to the Ref‐Seq mm9 annotation. To examine the associated gene sets, Gene Set Enrichment Analysis (GSEA) was conducted using the KEGG database.

### 2.16 Statistical analyses

All numerical information is presented in terms of the average ± standard deviation. Statistical evaluations were carried out utilising the GraphPad Prism (GraphPad Prism 8; GraphPad). Normality of comparisons was determined by Shapiro–Wilk (S–W) test. Comparisons between two groups employed unpaired *t*‐tests for statistical evaluation. Differences among multiple groups were evaluated utilising one‐way ANOVA and two‐way ANOVA.

## 3 RESULTS

### 3.1 *EEPD1* level elevated in human and murine atherosclerotic plaques

**FIGURE 1:**
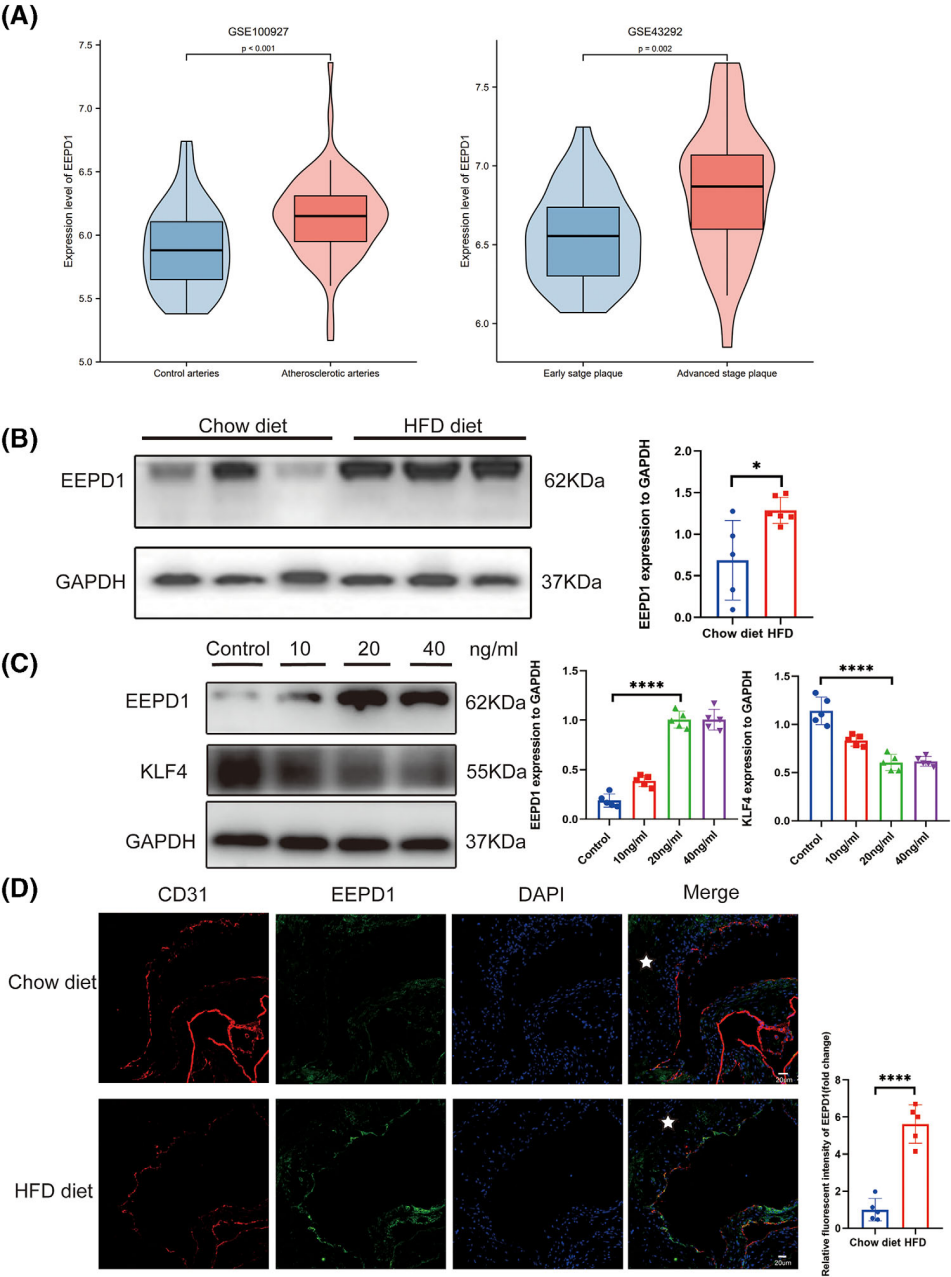
The *EEPD1* level elevated in human and murine atherosclerotic plaques. (A) Levels of *EEPD1* in control peripheral arteries without atherosclerotic lesions and arteries with atherosclerotic lesions based on the publicly available microarray databases GSE100927 (*n* = 35 for control arteries and *n* = 69 for atherosclerotic arteries) and GSE43292 (samples from carotid endarterectomy and advanced‐stage plaques defined as stage IV and over according to the Stary classification and early‐stage plaques defined as stages I and II; *n* = 32 per group). An unpaired Student's *t*‐test was used. (B) Representative immunoblots and summarised data showing HFD‐induced expression of EEPD1. Chow diet group *n* = 5, HFD group *n* = 6. (C) Representative immunoblots and summarised data showing TNFα‐induced expression of EEPD1 and KLF4 in HAECs. Each group *n* = 5. (D) Representative immunofluorescence pictures indicating increasing EEPD1 in endothelia. Chow diet group *n* = 5, HFD group *n* = 5. Scale bar = 20 µm.

To assess the involvement of EEPD1 in atherosclerosis, we analysed the expression of *EEPD1* mRNA expression in human atherosclerotic samples sourced from the NCBI Gene Expression Omnibus (NCBI/GEO). In the GSE100927 dataset, EEPD1 expression was found to be elevated in peripheral atherosclerotic arteries compared to control arteries without lesions (Figure 1A). Similarly, the GSE43292 dataset revealed increased *EEPD1* levels in carotid plaques compared to distant macroscopically intact tissue (Figure 1A). Moreover, the aorta of ApoE^–/–^ mice exhibited increased EEPD1 protein expression after a 12 weeks of HFD, compared to those fed a chow diet (Figure 1B). Significantly increased level of EEPD1 and decreased level of KLF4 was found in HAEC stimulated with TNFα (Figure 1C). In addition, *EEPD1* increased in the endothelium of aorta root sections of *ApoE* ^−/−^ mice after 12 weeks of HFD compared to chow diet, manifesting as a steady rise in fluorescence intensity (Figure 1D). The enhanced expression in atherosclerotic endothelium suggests a significant role of endothelial *EEPD1* in atherosclerosis progression.

### 3.2 *EEPD1* knockout ameliorated atherosclerosis in *ApoE* ^−/−^ hyperlipidemic mouse model

**FIGURE 2:**
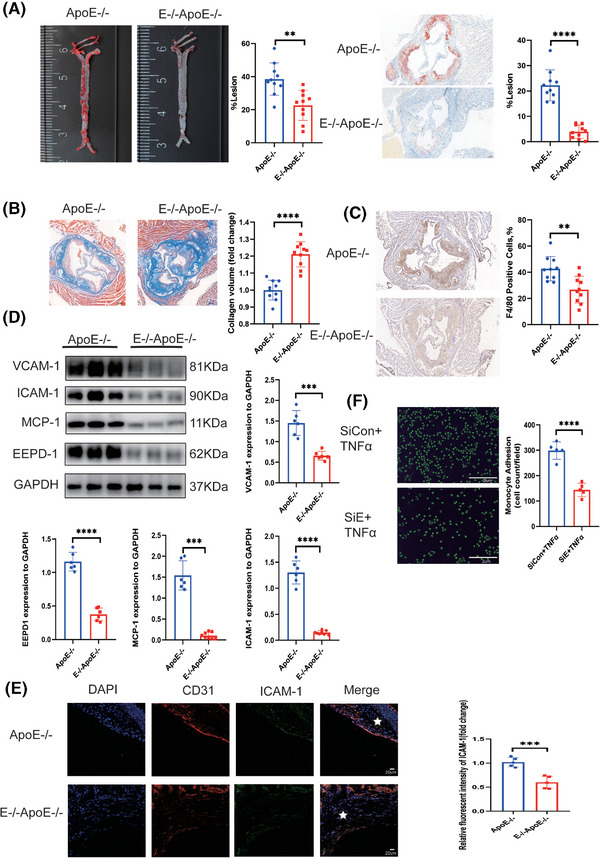
Knockout of *EEPD1* ameliorated atherosclerosis in *ApoE* ^–/–^ hyperlipidemic mouse model. (A) Representative Oil Red O staining of the entire aorta showing decreasing plaque area in E^–/–^ *ApoE* ^–/–^ mice compared to *ApoE* ^–/–^ mice. *n* = 10 per group. Representative images of Oil red O staining of the cross‐sections in the aortic sinus. *ApoE* ^–/–^ *n* = 10, E^–/–^ *ApoE* ^–/–^ *n* = 10. Scale bar = 200 µm. (B) Representative Masson staining showing collagen content of aortic plaques. *n* = 10 per group. Scale bar = 200 µm. (C) Representative F4/80 immunohistochemical staining. *ApoE* ^–/–^ *n* = 10, E^–/–^ *ApoE* ^–/–^ *n* = 10. (D) Representative immunoblots showing inhibition of VCAM‐1, ICAM‐1, MCP‐1 induced by EEPD1 knockout. (E) Representative immunofluorescence pictures showing inhibition of ICAM‐1 in endothelia induced by EEPD1 knockout. *n* = 5 per group. Scale bar = 20 µm. (F) HAEC were pretreated with scramble small‐interfering RNA (siRNA) or *EEPD1* siRNA followed by stimulation with TNFα as described above. Adhered CFSE‐stained THP‐1 cells were visualised by fluorescence microscopy and quantified. *n* = 5 per group. Scale bar = 2 µm. *p < .05, **p < .01, ***p < .001, ****p < .0001.

Since *EEPD1* levels were increased in the aortas of *ApoE* ^−/−^ mice after HFD for 12 weeks, we examined the role of *EEPD1* on atherosclerotic plaques. Then, *EEPD1* ^−/−^ *ApoE* ^−/−^ mice models were constructed by mating *EEPD1* ^−/−^ mice with *ApoE* ^−/−^ mice (Figure S1). A significant increase was observed in triglyceride and cholesterol concentrations in *EEPD1* ^−/−^ *ApoE* ^−/−^mice on HFD compared to *ApoE* ^−/−^ mice (Figure S2). And HE staining demonstrated that knockout of EEPD1 does not exert significant effects on other major organs under physiological conditions (Figure S3A‐E). We confirmed *EEPD1* protein level in multiple organs of *ApoE* ^−/−^ mice (Figure S3F). *EEPD1* deletion significantly decreased the atherosclerotic lesion area after 12 weeks of HFD as measured by Oil Red O staining compared to that of *ApoE* ^−/−^ mice (Figure 2A). Furthermore, collagen content was increased in *EEPD1* ^−/−^ *ApoE* ^−/−^ mice compared to *ApoE* ^−/−^ mice (Figure 2B). Also, F4/80^+^ region was reduced in *EEPD1* ^−/−^ *ApoE* ^−/−^ mice after 12 weeks of HFD (Figure 2C). Compared to WT *ApoE* ^−/−^ mice, protein levels of ICAM‐1, VCAM‐1 and MCP‐1 were markedly decreased in *EEPD1* ^−/−^ *ApoE* ^−/−^ mice (Figure 2D). Immunofluorescence staining revealed that ICAM‐1 decreased after Edpd1 knockout (Figure 2E), and *EEPD1* siRNA transfection suppressed the attachment of THP‐1 cells onto HAEC activated by TNFα (Figure 2F).

### 3.3 *EEPD1* knockout mitigated endothelial apoptosis in atherosclerosis

**FIGURE 3:**
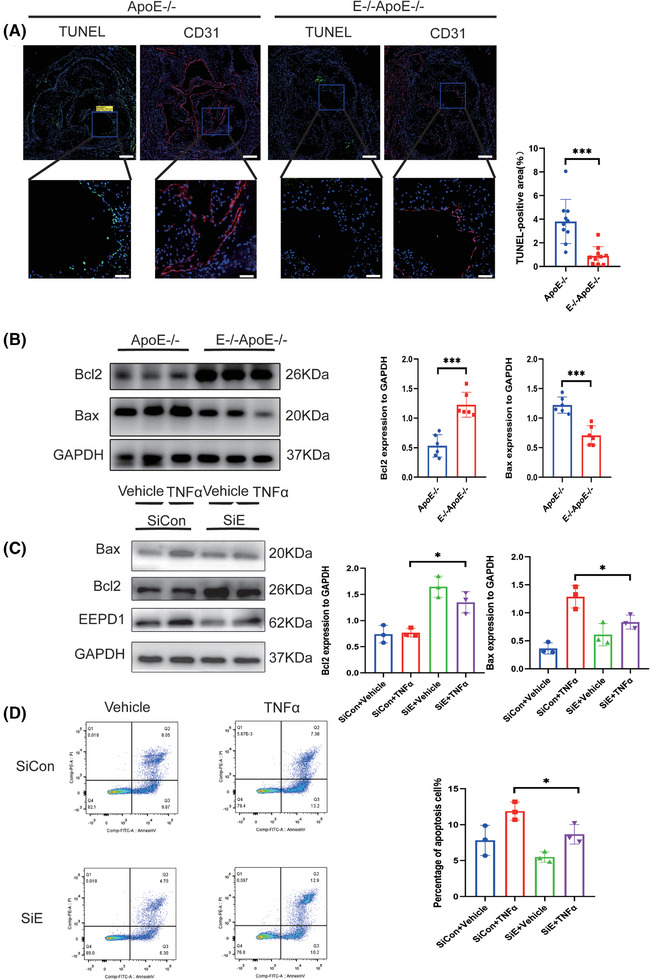
Knockout of *EEPD1* mitigated the apoptosis of endothelial in atherosclerosis. (A) Representative TUNEL and CD31 staining. Scale bar = 200 µm (upper); 50 µm (lower). (B) Representative immunoblots showing changes in protein levels of Bcl2 and Bax induced by EEPD1 knockout. (C) Representative immunoblots showing changes in protein levels of Bcl2 and Bax in HAECs. (D) Representative flow cytometric plot showing percentage of apoptosis HAECs in each group. *n* = 3.

Endothelial apoptosis is a pivotal factor in the progression of atherosclerosis. As shown in Figure 3A, deletion of *EEPD1* decreased the TUNEL‐positive area while Bax level declined and Bcl2 level rose in *EEPD1* ^−/−^ *ApoE* ^−‐/−^ mice compared to that in *ApoE* ^−/−^ mice (Figure 3B). Then, we probed how EEPD1 knockout affects TNFα‐induced endothelial injury in vitro. *EEPD1* siRNA transfection significantly reduced *EEPD1* protein expression (Figure 3C). *EEPD1* siRNA transfection significantly decreases Bax and elevates Bcl2 protein levels (Figure 3C). Analysis of HAECs by flow cytometry revealed that *EEPD1* siRNA transfection group has lower apoptosis rate than negative siRNA transfection group after TNFα stimulation (Figure 3D).

### 3.4 *EEPD1* overexpression exacerbated the progression of atherosclerosis in *ApoE* ^−/−^ mice

**FIGURE 4:**
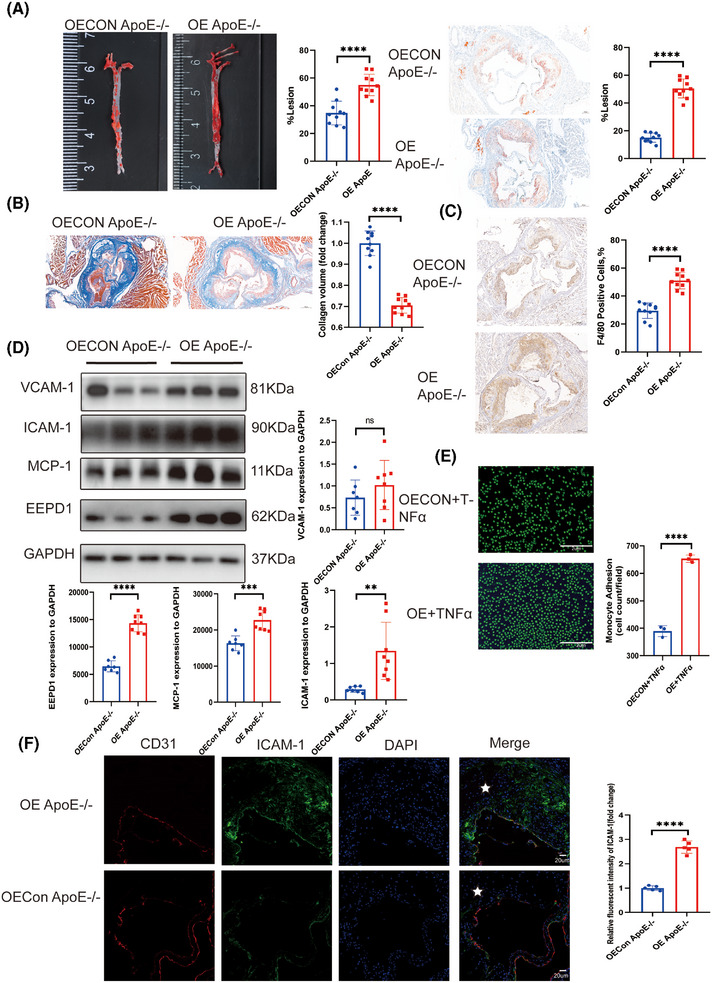
*EEPD1* overexpression exacerbated the progression of atherosclerosis in ApoE^−/−^ mice. (A) Representative Oil Red O staining of the entire aorta showing increasing plaque area in OE*ApoE* ^–/–^ mice compared to OECON*ApoE* ^–/–^ mice. *n* = 10 per group. Scale bar = 200 µm. (B) Representative Masson staining showing collagen content of aortic plaques. *n* = 10 per group. Scale bar = 200 µm. (C) Representative F4/80 immunohistochemical staining. *n* = 10 per group. Scale bar = 200 µm. (D) Representative immunoblots showing increase of VCAM‐1, ICAM‐1, MCP‐1 induced by EEPD1 overexpression. (E) HAEC were pretreated with scramble small‐interfering RNA (siRNA) or *EEPD1* siRNA followed by stimulation with TNFα as described above. Adhered CFSE‐stained THP‐1 cells were visualised by fluorescence microscopy and quantified. OECon *n* = 3, OE *n* = 4. Scale bar = 2 µm. (F) Representative immunofluorescence pictures showing increase of ICAM‐1 in endothelia induced by EEPD1 overexpression. *n* = 5 per group. Scale bar = 20 µm.

To further investigate function of *EEPD1*, *EEPD1* overexpression vector‐injected model was constructed. No significant differences were detected in blood lipid levels between the OEApoE^–/–^ mice on HFD and the OEConApoE^–/–^ mice (Figure S2). Atherosclerotic lesions were visualised with Oil Red O staining (Figure 4A), revealing that injection of *EEPD1* overexpression vector significantly increased atherosclerotic lesion area after 12 weeks of HFD compared to the control group. Furthermore, collagen content declined in *EEPD1* overexpression vector injected mice compared to control mice (Figure 4B). In addition, F4/80^+^ area was larger in *EEPD1* overexpression injected mice after 12 weeks of HFD (Figure 4C). The upregulation of *EEPD1* protein level in the aortas of *ApoE* ^−/−^ mice after HFD for 12 weeks was verified by Western blot (Figure 4D). Moreover, although the level of VCAM‐1 protein was upregulated in the EEPD1 overexpression vector‐injected mice, this increase was not statistically significant, whereas ICAM‐1 protein was significantly upregulated (Figure 4D). In addition, transfection of *EEPD1* overexpressed plasmid increased the attachment of monocytic THP‐1 cells to TNFα‐activated HAECs (Figure 4E), which was in agreement with our in vivo results. Immunofluorescence staining revealed that endothelial ICAM‐1 increased after *EEPD1* overexpression (Figure 4F).

### 3.5 *EEPD1* overexpression aggravated the apoptosis of endothelial cells in atherosclerosis

**FIGURE 5:**
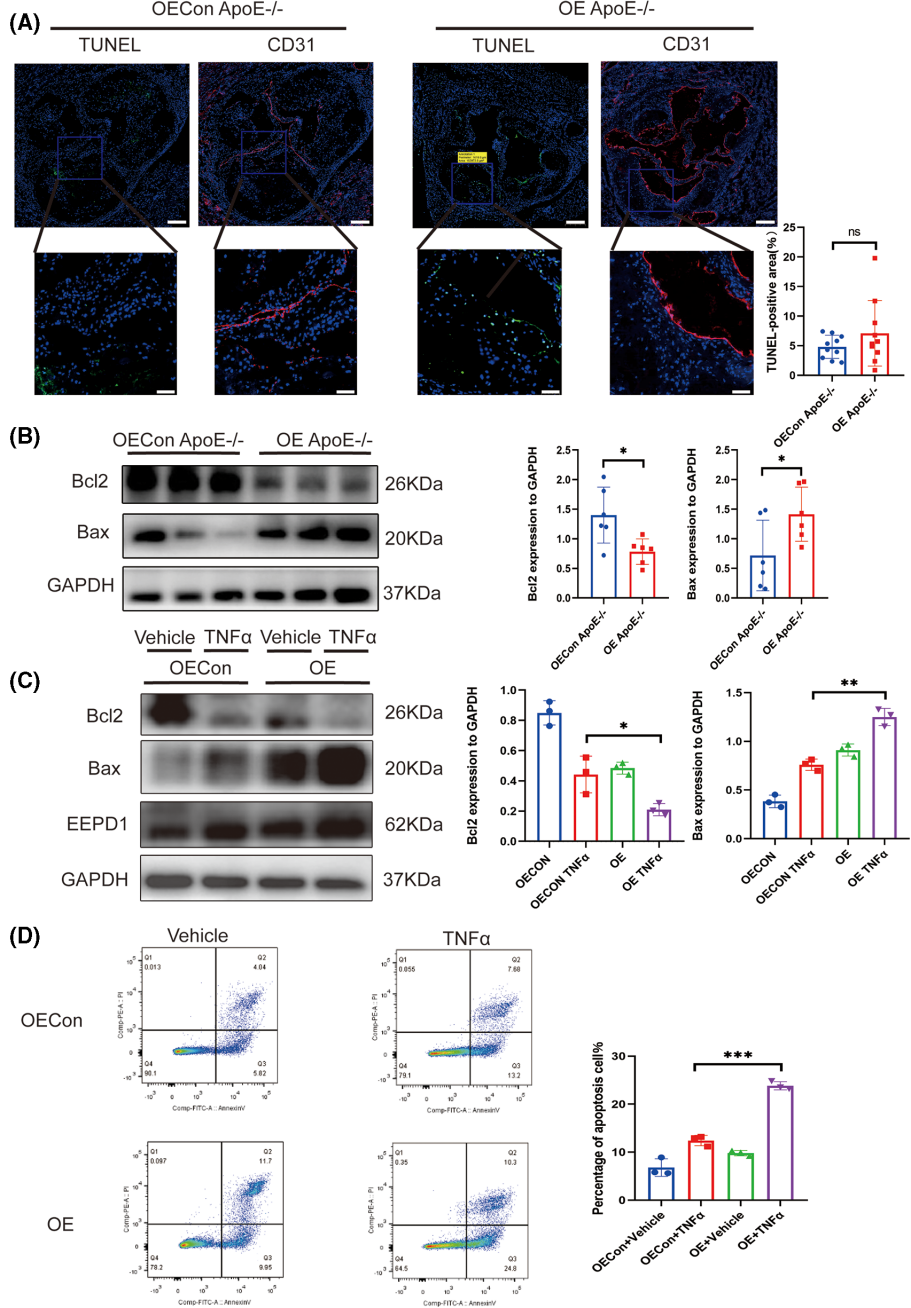
EEPD1 overexpression aggravated the apoptosis of endothelial in atherosclerosis. (A) Representative TUNEL and CD31 staining. OEConApoE^–/–^ *n* = 10, OEApoE^–/–^ *n* = 10. Scale bar = 200 µm (upper); 50 µm (lower). (B) Representative immunoblots showing changes in protein levels of Bcl2 and Bax induced by EEPD1 overexpression in vivo. (C) Representative immunoblots showing changes in protein levels of Bcl2 and Bax induced by EEPD1 overexpression in vitro. (D) Representative flow cytometric plot showing percentage of apoptosis HAECs in each group. *n* = 3.

Subsequently, we probed the impact of *EEPD1* on endothelial apoptosis in atherosclerosis. An increased trend in TUNEL‐positive area in *EEPD1* overexpression vector injected mice compared to the empty vector injected group, although the difference didn't attain statistical significance (Figure 5A). Then, compared to empty vector injected mice, Bax protein level elevated and Bcl2 protein level reduced in *EEPD1* overexpression vector injected mice (Figure 5B). *EEPD1* overexpression plasmid transfection significantly elevated Bax and decreased Bcl2 protein levels (Figure 5C). In addition, flow cytometry results showed that the transfection of *EEPD1* overexpressed plasmid significantly elevated the apoptosis of HAECs (Figure 5D).

### 3.6 Activation of *ERK* phosphorylation abrogated the protective effect of *EEPD1* knockout in endothelium in atherosclerosis

**FIGURE 6:**
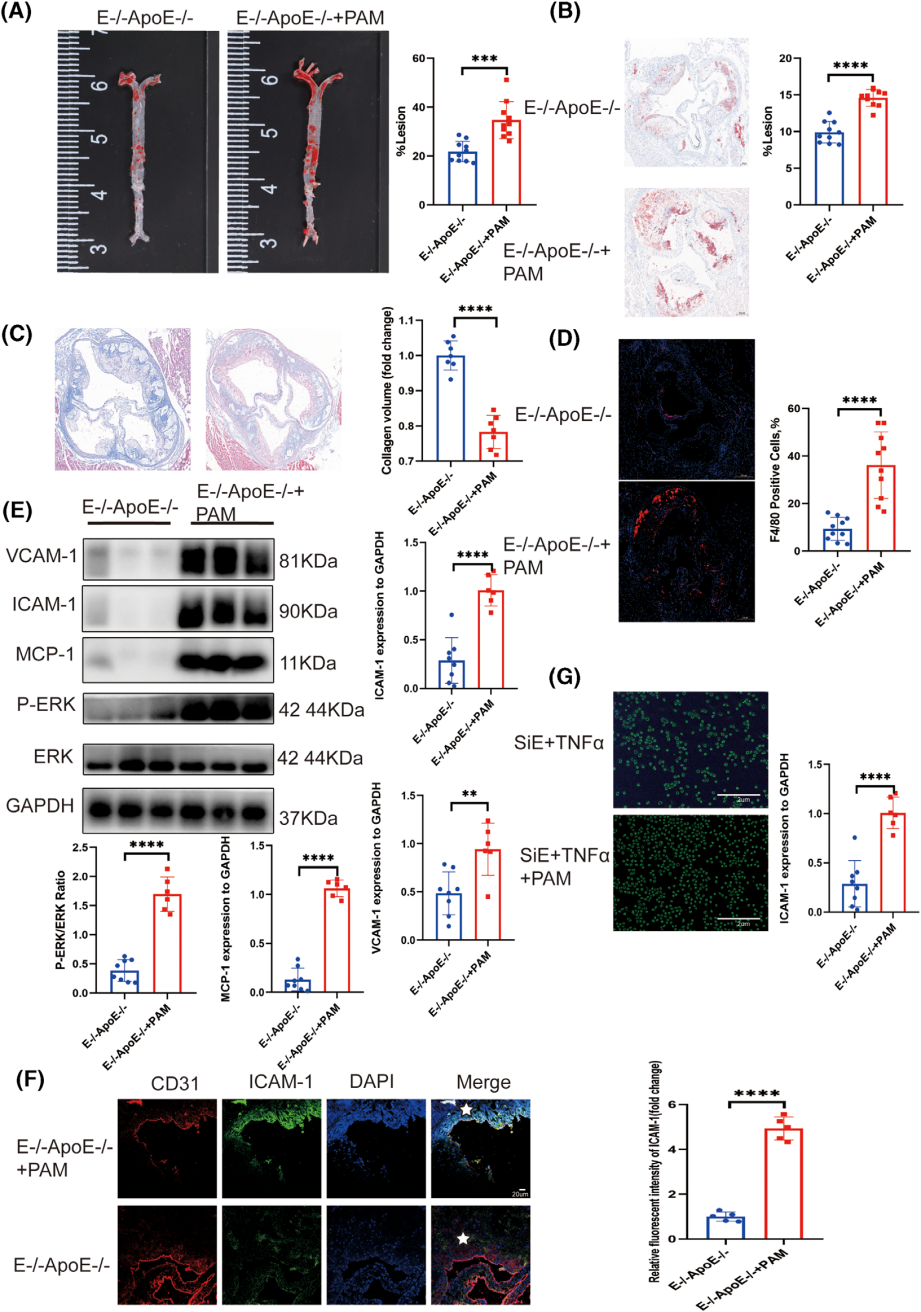
Activation of *ERK* phosphorylation abrogated the protective effect of *EEPD1* knockout on endothelium in atherosclerosis. (A) Representative Oil Red O staining of the entire aorta showing increasing plaque area in E^–/–^ *ApoE* ^–/–^ +PAM mice compared to E^–/–^ *ApoE* ^–/–^ mice. *n* = 10. (B) Representative images of Oil red O staining of the cross‐sections in the aortic sinus. *n* = 10 per group. Scale bar = 200 µm. (C) Representative Masson staining showing collagen content of aortic plaques. *n* = 10 per group. Scale bar = 200 µm. (D) Representative F4/80 immunohistochemical staining. E^–/–^ApoE^–/–^ *n* = 10, E^–/–^ApoE^–/–^+PAM *n* = 10. Scale bar = 200 µm. (E) Representative immunoblots showing increase of VCAM‐1, ICAM‐1, MCP‐1 induced by ERK phosphorylation activation in E^–/–^ApoE^–/–^ mice. (F) Representative immunofluorescence pictures showing increase of ICAM‐1 in endothelia induced by *ERK* phosphorylation activation in E^–/–^ApoE^–/–^ mice. Scale bar = 20 µm. (G) HAEC were pretreated with scramble small‐interfering RNA (siRNA) or *EEPD1* siRNA followed by stimulation with TNFα and pamoic acid as described above. Adhered CFSE‐stained THP‐1 cells were visualised by fluorescence microscopy and quantified. *n* = 3 per group. Scale bar = 2 µm.

To elucidate the protective mechanism of EEPD1 knockout on endothelial cells in the context of atherosclerosis, we conducted high‐throughput RNA sequencing (RNA‐seq) across four experimental groups: siCon, siCon+TNFα, siEEPD1, and siEEPD1+TNFα. The results of GO enrichment showed that it was mainly enriched in protein phosphorylation pathway between siCon+TNFα and si*EEPD1*+TNFα groups (Figure S4A). The results of KEGG enrichment showed that *EEPD1* was mainly enriched in the MAPK pathway between the siCon+TNFα and si*EEPD1*+TNFα groups (Figure S4B). The enriched pathways from Reactome include PECAM‐1 interaction (Figure S4C and D). The volcano plot indicated that MAPK1 is downregulated in the si*EEPD1*+TNFα group (Figure S4E). Furthermore, the Venn diagram illustrates that the alterations in MAPK1 mRNA levels are attributable to the knockout of EEPD1, rather than being elicited by the action of TNFα (Figure S4F). Using Western blot, we assessed the phosphorylation levels of P38, JNK, ERK, and observed an increase in ERK phosphorylation following EEPD1 knockdown, whereas insignificant alterations were noted in the phosphorylation of JNK and P38 (Figure S4G). Subsequently, we explored the relationship between *EEPD1* and *ERK* phosphorylation. Activation of the ERK signalling pathway was achieved through intraperitoneal injection of pamoic acid. Notably, no significant difference was detected in blood lipid levels in *EEPD1* ^−/−^ *ApoE^−/−^* mice injected with pamoic acid compared to *EEPD1* ^−/−^ *ApoE* ^−/−^ mice injected with saline (Figure S2). Surprisingly, the atherosclerotic lesion area increased significantly in *EEPD1* ^−/−^ *ApoE* ^−/−^ mice injected pamoic acid compared to those injected with saline after 12 weeks of HFD (Figure 6A and B). Furthermore, the collagen content was decreased in pamoic acid‐injected *EEPD1* ^−/−^ *ApoE* ^−/−^ mice compared to saline‐injected *EEPD1* ^−/−^ *ApoE* ^−/−^ mice (Figure 6C). The F4/80^+^ stained region was larger in *EEPD1* ^−/−^ *ApoE* ^−/−^ mice injected pamoic acid after 12 weeks of HFD (Figure 6D). Compared to *EEPD1* ^−/−^ *ApoE* ^−/−^ mice injected saline, the protein levels of ICAM‐1, VCAM‐1 and MCP‐1 were markedly increased in *EEPD1* ^−/−^ *ApoE* ^−/−^ mice injected pamoic acid (Figure 6E). Immunofluorescence staining revealed that VCAM‐1 was upregulated after pamoic injection (Figure 6F). In vitro, pamoic treatment significantly abrogated the adhesion of THP‐1 cells onto TNFα‐stimulated HAEC monolayers induced by *EEPD1* siRNA transfection (Figure 6G).

### 3.7 Activation of *ERK* phosphorylation abrogated the suppressive effect of *EEPD1* knockout in endothelial apoptosis in atherosclerosis

**FIGURE 7:**
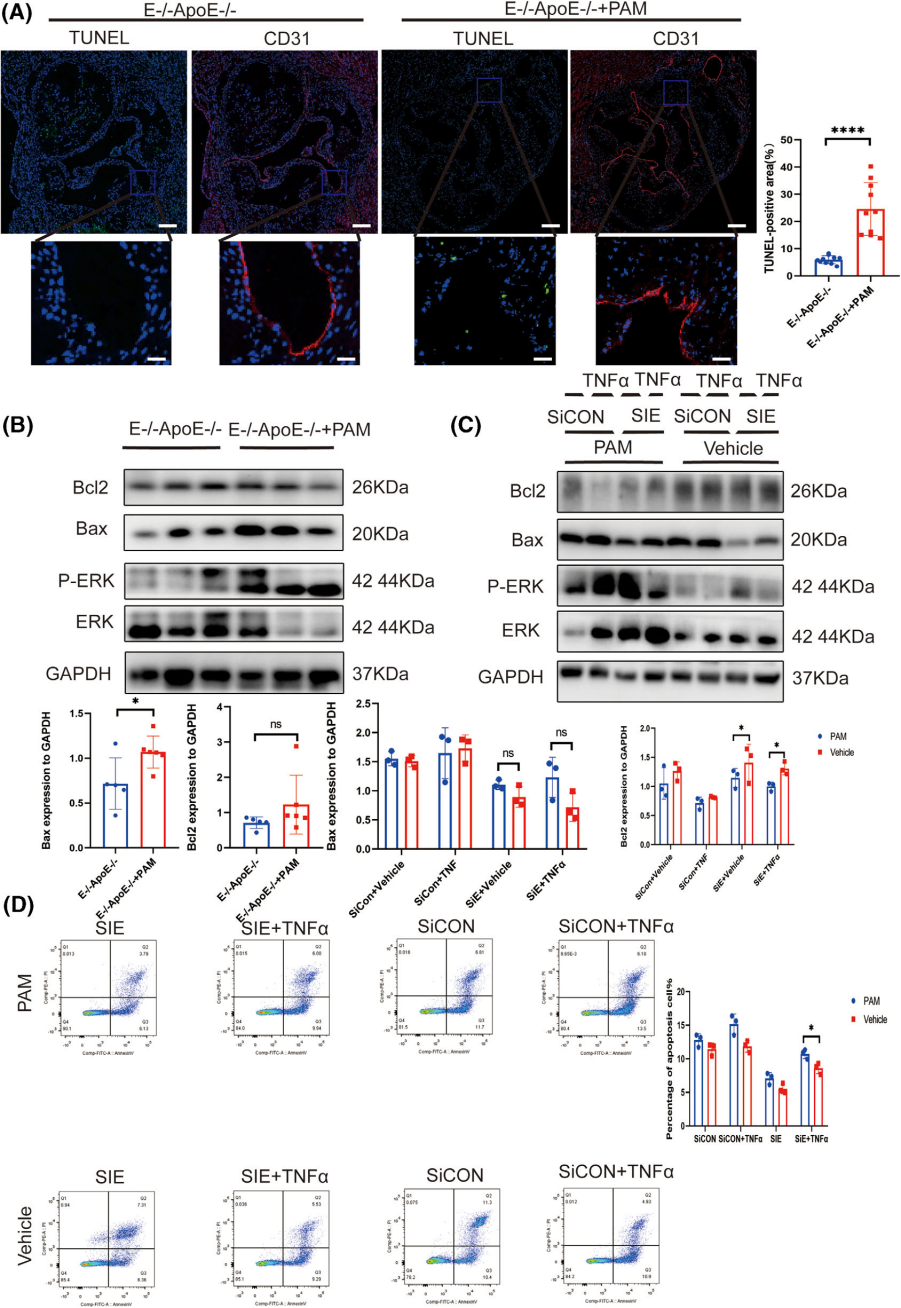
Activation of *ERK* phosphorylation abrogated the suppressive effect of *EEPD1* knockout on endothelial apoptosis in atherosclerosis. (A) Representative TUNEL and CD31 staining. *n* = 5. Scale bar = 200 µm (upper); 50 µm (lower). (B) Representative immunoblots showing changes in protein levels of Bcl2 and Bax induced by *ERK* phosphorylation activation in E^–/–^ApoE^–/–^ mice. (C) Representative immunoblots showing changes in protein levels of Bcl2 and Bax in HAECs. (D) Representative flow cytometric plot showing percentage of apoptosis HAECs in each group.

Next, we probed whether *EEPD1* regulates endothelial apoptosis through *ERK* signalling pathway. Pamoic acid injection significantly increased the TUNEL‐positive area in *EEPD1* ^−/−^ *ApoE* ^−/−^ mice compared to saline injection (Figure 7A). In addition, Bax protein level increased while Bcl2 level showed no difference in pamoic acid‐injected *EEPD1* ^−/−^ *ApoE* ^−/−^ mice compared to saline‐injected *EEPD1* ^−/−^ *ApoE* ^−/−^ mice (Figure 7B). Consistent with the in vivo results, pamoic acid treatment abrogated the down‐ and upregulation of Bax and Bcl2 proteins induced by *EEPD1* siRNA transfection in HAECs (Figure 7C). Flow cytometry results revealed that *EEPD1* siRNA transfection‐induced apoptosis of HAECs was abrogated by pamoic acid treatment (Figure 7D).

### 3.8 Inhibition of *KLF4* abrogated the protective effect of *EEPD1* knockout in the endothelium in atherosclerosis

**FIGURE 8:**
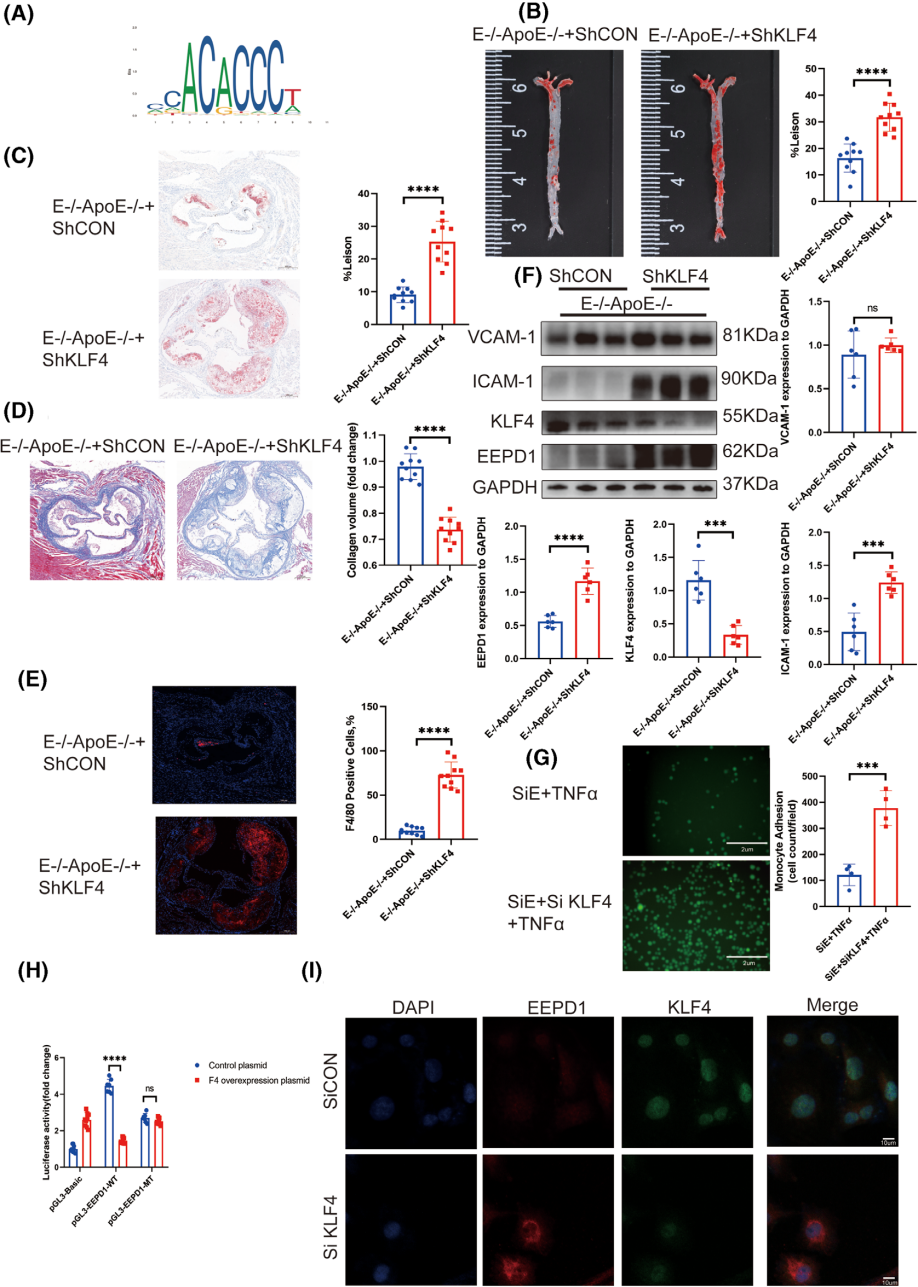
Inhibition of *KLF4* abrogated the protective effect of *EEPD1* knockout on endothelium in atherosclerosis. (A) Predicted region in Jaspar. (B) Representative Oil Red O staining of the entire aorta showing increasing plaque area in E^–/–^ *ApoE* ^–/–^ +ShKLF4 mice compared to E^–/–^ *ApoE* ^–/–^ mice. *n* = 7. (C) Representative images of Oil red O staining of the cross‐sections in the aortic sinus. E^–/–^ *ApoE* ^–/–^+Vehicle *n* = 10, E^–/–^ *ApoE* ^–/–^+ShKLF4 *n* = 11. Scale bar = 200 µm. (D) Representative Masson showing collagen content of aortic plaques. *n* = 7 per group. Scale bar = 200 µm. (E) Representative F4/80 immunohistochemical staining. *n* = 5 per group. Scale bar = 200 µm. (F) Representative immunoblots showing increase of VCAM‐1, ICAM‐1, MCP‐1 induced by KLF4 knockdown in E^–/–^ApoE^–/–^ mice. (G) HAEC were pretreated with *EEPD1* siRNA followed by stimulation with TNFα or TNFα plus *KLF4* inhibitor as described above. Adhered CFSE‐stained THP‐1 cells were visualised by fluorescence microscopy and quantified. *n* = 3 per group. Scale bar = 2 µm. (H) Result of luciferase assay. (I) Representative immunofluorescence pictures of HAECs. Scale bar = 10 µm.

After insertion of the *EEPD1* promoter region into JASPAR, we identified several transcription factors as potential upstream regulators of *EEPD1*, including *KLF4* (Figure 8A). No significant difference was detected in triglyceride and cholesterol concentrations in *EEPD1* ^−/−^ *ApoE^−/−^* mice injected AAV9‐hFLT1‐ShKLF4 or empty AAV9 vector (Figure S2D). Compared to injection of AAV9‐ hFLT1‐ShCON, an increase in atherosclerotic plaque area positive area was observed in EEPD1^–/–^ApoE^–/–^ mice after injection of AAV9‐hFLT1‐ShKLF4 (Figure 8B and C). Furthermore, knockdown of KLF4 reduced the collagen content while increasing the F4/80+ positive area in *EEPD1* ^−/−^ *ApoE* ^−/−^ (Figure 8D and E). Protein levels of VCAM‐1 increased after KLF4 knockdown in *EEPD1* ^−/−^ *ApoE* ^−/−^ mice, but not significantly. And ICAM‐1 level rose after KLF4 knockdown in *EEPD1* ^−/−^ *ApoE* ^−/−^ mice (Figure 8F). Decrease in VCAM‐1, ICAM‐1, and MCP‐1 protein levels after *EEPD1* siRNA transfection was abolished by KLF4 knockdown in HAECs (Figure S5A), while the mRNA (Figure S5B) level of *EEPD1* increased after *KLF4* was suppressed. KLF4 knockdown significantly exacerbated the adhesion of THP‐1 cells onto TNα‐stimulated HAEC monolayers induced by *EEPD1* siRNA transfection (Figure 8G). Next, we conducted ChIP assays and found a direct association of *KLF4* with *EEPD1*; this finding indicated that *EEPD1* is a direct target gene of *KLF4* (Figure S5C). Compared to mutant *EEPD1* plasmid, the WT plasmid lost the luciferase activity under *KLF4* overexpression (Figure 8H). To investigate spatial correlation between *KLF4* and *EEPD1*, we utilised immunofluorescence. Results indicated that KLF4 primarily distributed in the nucleus of HAECs, whereas EEPD1 predominantly localised in the cytoplasm. And the fluorescence of *EEPD1* increased after *KLF4* knockdown (Figure 8I).

### 3.9 Inhibition of *KLF4* abolished the suppressive effect of *EEPD1* knockout in endothelial apoptosis in atherosclerosis

**FIGURE 9:**
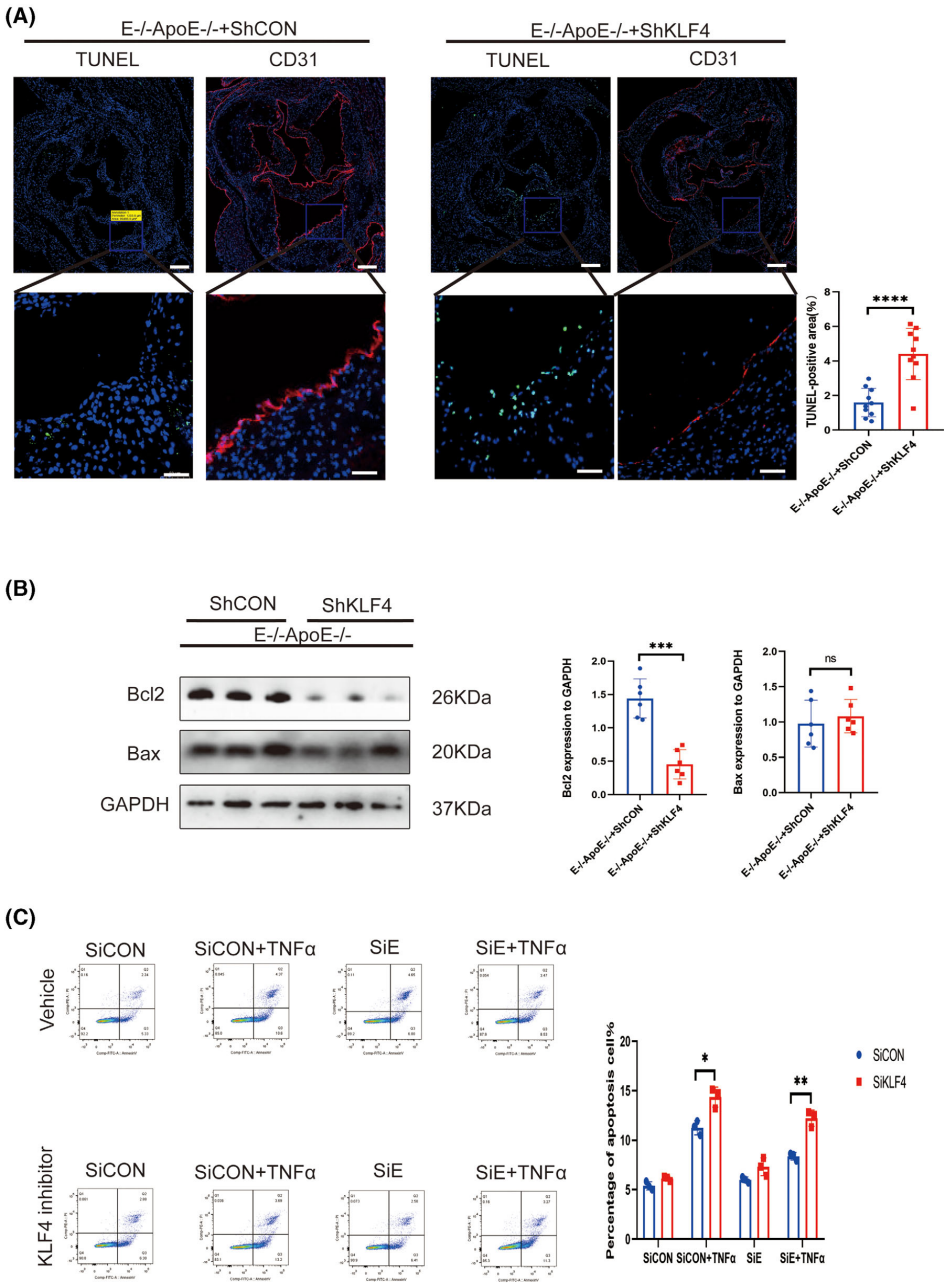
Inhibition of *KLF4* abolished the suppressive effect of *EEPD1* knockout on endothelial apoptosis in atherosclerosis. (A) Representative TUNEL and CD31 staining(*n* = 5). Scale bar = 20 µm (upper); 50 µm (lower). (B) Representative immunoblots showing changes in protein levels of Bcl2 and Bax induced by KLF4 knockdown in E^–/–^ApoE^–/–^ mice. (*n* = 6). (C) Representative flow cytometric plot showing percentage of apoptosis HAECs in each group.

Furthermore, we explored the regulatory role of *KLF4* upstream of *EEPD1*. AAV9‐hFLT1‐ShKLF4 injection significantly increased the TUNEL‐positive area of *EEPD1* ^−/−^ *ApoE* ^−/−^ mice compared to empty AAV9 vector injection (Figure 9A). Then, Bax protein level slightly increased in AAV9‐hFLT1‐ShKLF4 injected *EEPD1* ^−/−^ *ApoE* ^−/−^ mice compared to empty AAV9 vector‐injected *EEPD1* ^−/−^ *ApoE* ^−/−^ mice, but not significantly, while significantly decrease was observed in Bcl2 across the two groups (Figure 9B). Consistent with the in vivo results, Flow cytometry showed that *EEPD1* siRNA transfection elevated the apoptosis levels of HAECs that were abrogated by KLF4 knockdown (Figure 9C).

## 4 DISCUSSION

Atherosclerosis represents a chronic condition marked by the gradual build‐up of fatty substances and fibrous components within major arteries. The complex responses are associated with endothelial inflammation, excessive lipid deposition, and foamy cell formation. However, the underlying mechanisms remain largely unknown.

During the process of atherosclerosis, endothelial activation is crucial to monocyte recruitment.^28^ Interestingly, *EEPD1* is upregulated in embryonic stem cells in response to DNA damage. Moreover, *EEPD1* ameliorates atherosclerosis by promoting cholesterol efflux through the LXR‐*EEPD1*‐ABCA1/G1 pathway.^29^, ^30^ Despite these findings, sufficient in vivo evidence for EEPD1's role in atherogenesis is still lacking, and the mechanisms by which EEPD1 influences endothelial inflammation and apoptosis in atherosclerosis are not yet fully elucidated. Our research indicates that EEPD1 exacerbates vascular inflammation and apoptosis by modulating ERK phosphorylation in endothelial cells, thereby promoting monocyte adhesion, migration, and penetration into the vascular wall. This process contributes to the formation of lipid‐laden foam cells, thereby initiating atherosclerosis. It is notable that EEPD1 is essential to regulating cholesterol metabolism in macrophages, and knocking out EEPD1 in macrophages may result in an elevation of cholesterol levels. Consequently, when considering EEPD1 as a target for clinical translation and intervention in atherosclerosis, its vascular endothelial specificity is of paramount importance. In our study, we used global EEPD1 knockout mice. Although EEPD1 was knocked out in macrophages, the overall phenotype suggested that the beneficial effects of endothelial EEPD1 knockout on atherosclerosis outweighed the adverse effects in macrophages.

The gene expression in the MAPK pathway participates in cell proliferation,^31^ motility,^32^ adhesion,^33^ apoptosis,^34^ and glucose metabolism.^18^ Upon activation, ERK migrates to the nucleus where it phosphorylates numerous substrates, encompassing transcription factors like CREB and Elk1. A study using *GPR146* ^−/−^ *LDLR* ^−/−^ mice confirmed that GPR146 regulates plasma cholesterol levels and the course of atherosclerosis through *ERK* signalling.^35^ The results of our study indicated that EEPD1 expression is increased in atherosclerotic endothelium, and the absence of EEPD1 in endothelial cells reduces the development of atherosclerotic lesions, suggesting a new function for endothelial EEPD1 in the process of atherogenesis. The current study indicated that absence of endothelial *EEPD1* reduces monocyte adherence to the vascular wall and the formation of proinflammatory macrophages, as a result of inhibited *ERK* phosphorylation in ECs.

Currently, the pharmacological management of atherosclerosis primarily includes the following categories of drugs: antiplatelet agents and oral anticoagulants, beta blockers, renin‐angiotensin‐aldosterone inhibitors, colchicine, medical therapy for relief of angina, management of refractory angina, chelation therapy.^36^ Colchicine exerts anti‐inflammatory effects by modulating inflammatory cell‐mediated chemotaxis and phagocytosis through the inhibition of microtubule polymerisation. Additionally, colchicine also reduces the expression of adhesion molecules and influences cytokine production. However, the narrow therapeutic index and the propensity for drug–drug interactions associated with Colchicine have constrained its clinical application. This study suggests EEPD1 as a promising avenue for creating new therapeutic agents, possibly with anti‐tumour effects. Consequently, EEPD1 targeting may be more appropriate for atherosclerotic patients who require polypharmacy, particularly those with concurrent tumours, as opposed to colchicine, which is susceptible to drug–drug interactions.

In addition to atherosclerosis, targeting EEPD1 may also have an impact on other cardiovascular diseases. Knocking out EEPD1 can reduce endothelial apoptosis, potentially promoting endothelial cell proliferation and enhancing endothelial barrier function, which is indeed beneficial for relieving atherosclerosis. However, excessive proliferation of vascular smooth muscle and endothelium is an important cause of pulmonary hypertension.^37^, ^38^, ^39^ Although we may later target the EEPD1 of vascular endothelial cells for knockout through endothelial markers to avoid affecting vascular smooth muscle, excessive proliferation of endothelial cells is indeed a factor that needs to be considered for clinical application. Some studies have shown that the enhancement of endothelial cell barrier function can inhibit onset and progression of abdominal aortic aneurysms.^40^ Therefore, targeting endothelial EEPD1 knockout also has the potential for prevention and treatment of abdominal aortic aneurysms. Previous study has also revealed that EEPD1 in the myocardium exerts protective effect against radiation‐induced cardiomyopathy through the EEPD1‐FOXO3A axis.^41^ EEPD1 exerts protective effect against radiation‐induced cardiomyopathy by modulating the ubiquitination level of FOXO3A through its interaction with FOXO3A, thereby regulating the protein stability of FOXO3A. It is well‐recognised that the same gene often plays distinct roles in different tissues and cells within the human body, and even at various stages of the same disease. The detrimental role of EEPD1 in atherosclerosis and its protective effect in radiation‐induced cardiomyopathy are not contradictory. This is because atherosclerosis is primarily driven by inflammation and mechanical stress in the vascular endothelium, whereas radiation‐induced cardiomyopathy is primarily driven by oxidative stress, DNA damage, and other factors resulting from radiation. There are clear differences in their driving mechanisms. Furthermore, the sites of action also differ, with one being the arterial vascular endothelium and the other being the cardiac myocardium. We also believe that targeting EEPD1 for the treatment of atherosclerosis necessitates a strong focus on the vascular endothelium, and that intervention at a relatively early stage of atherosclerosis may yield better outcomes.

Branches and curved regions of the arterial tree with disrupted blood flow are the preferred locations for atherosclerotic lesions.^42^ KLF4 exhibits elevated expression levels within the endothelium, though not consistently so. Specifically, it is notably expressed in the linear portions of the human aorta, whereas its expression is considerably diminished at branch points.^43^ Our findings revealed an opposite trend of *KLF4* and *EEPD1* in atherosclerotic lesions. The ChIP and luciferase assay results verified the interplay between KLF4 and EEPD1.

Nevertheless, the present study has some limitations: (1) We didn't generate endothelial‐specific *EEPD1* knockout mice and can't fully rule out the systemic effects from other organs affecting the progression of atherosclerosis. (2) Our study has not yet conducted a comprehensive examination of the precise mechanism through which EEPD1 modulates ERK1/2 phosphorylation. Our future research will involve the generation of endothelial‐specific EEPD1 knockout mice, with the aim of delving deeper into the mechanism by which EEPD1 regulates ERK1/2 phosphorylation. (3) In this study, pamoic acid was used as a nonspecific agonist for ERK1/2 phosphorylation, as specific ERK1/2 agonists are not commercially available.

Several challenges currently exist in targeting EEPD1 for the treatment of atherosclerosis. Firstly, there is a lack of small‐molecule inhibitors specific to EEPD1. With the increasing popularity of AlphaFold, screening for molecules that interact with EEPD1 is not difficult. However, subsequent extensive animal and clinical trials are necessary to verify effectiveness and safety. Secondly, the endothelial targeting specificity of the drug within arteries is also crucial, and potential off‐target effects necessitate a high degree of vigilance.

Taken together, this study provided novel evidence that endothelial *EEPD1* is directly regulated by the atheroprotective gene *KLF4*. Inflammation downregulates endothelial *KLF4* and upregulates *EEPD1* expression, stimulating *ERK* phosphorylation and promoting VCAM‐1, ICAM‐1, and MCP‐1 secretion. These phenomena impair the endothelial function and promote monocyte adhesion and infiltration into the vessel wall for the formation of proinflammatory foam cells and atherosclerotic lesions. Moreover, *KLF4*‐*EEPD1*‐*ERK* pathway could be the potential therapeutic target against atherosclerosis.

## 5 CONCLUSION

Overall, the results of our experiments demonstrated that harmful effects of *EEPD1* is directly regulated by the atheroprotective gene *KLF4*. And EEPD1 aggravated endothelial inflammation and apoptosis by regulating ERK phosphorylation. Thus, potential therapeutics that target KLF4‐EEPD1‐ERK axis in endothelia may be a new strategy for the prevention and treatment of atherosclerosis

## AUTHOR CONTRIBUTIONS

Conceptualisation, Ruogu Li and Min Zhang; Data curation, Kaiwen Yu; Formal analysis, Xiang Li and Xin Shi; Funding acquisition, Min Zhang, Ruogu Li, Xin Shi; Investigation, Kaiwen Yu; Methodology, Kaiwen Yu, Xiang Li; Resources, Kaiwen Yu and Min Zhang; Supervision, Ruogu Li and Min Zhang; Writing—original draft, Kaiwen Yu; Writing—review & editing, Xiang Li.

## CONFLICT OF INTEREST STATEMENT

The authors declare that they have no competing interests.

### ETHICS STATEMENT

The animal study was reviewed and approved by Shanghai Jiao Tong University Animal Care Committee and the Institute's Animal Ethics Committee of Shanghai Chest Hospital, Shanghai Jiao Tong University (approval number: KS(Y)24300). Consent to participate is not applicable.

## Supporting information



Supporting Information

## Data Availability

The datasets used or analysed during the current study are available from the corresponding author upon reasonable request.
