# Supplementary material for: EEPD1 regulates inflammation and endothelial apoptosis in atherosclerosis through KLF4‐EEPD1‐ERK axis
Source: Clin Transl Med. 2025 Apr 23;15(4):e70311. doi: 10.1002/ctm2.70311 (PMC12017893; doi:10.1002/ctm2.70311)
Supplement: Supplementary file 1 — Supporting Information [file CTM2-15-e70311-s001.docx]

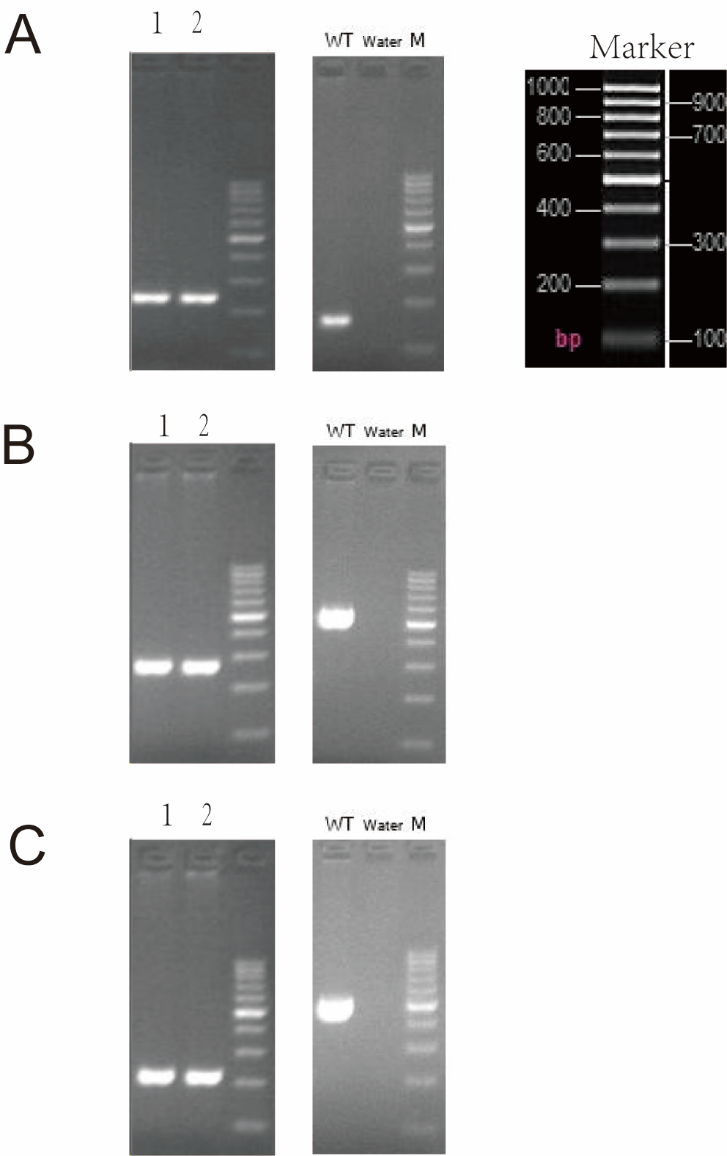


**Supplementary Figure1 A. *ApoE* PCR. Homozygotes: one band with 245 bp. Wildtype allele: 155 bp. B.** ***EEPD1* Region1 PCR.** **Homozygotes: one band with ~300 bp. Wildtype allele: 580 bp. C.** ***EEPD1* Region2 PCR.** **Homozygotes: one band with ~200 bp. Wildtype allele: 529 bp.**


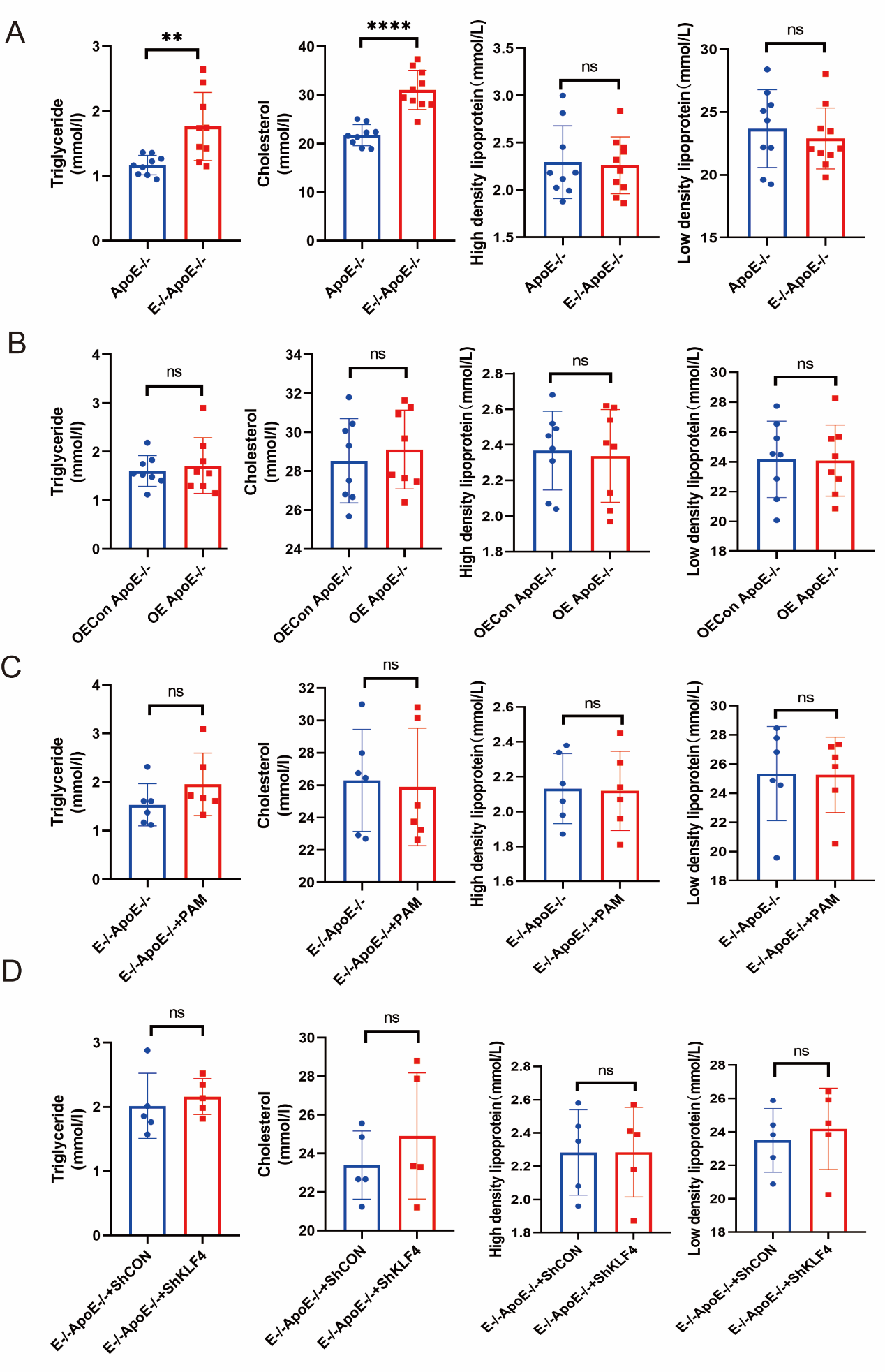


**Supplementary Figure2 A. Plasma triglyceride, cholesterol, high density lipoprotein and low density lipoprotein levels of *ApoE*-/- and E-/-*ApoE*-/- mice. B. Plasma triglyceride, cholesterol, high density lipoprotein and low density lipoprotein levels of OECon *ApoE*-/- and OE *ApoE*-/- mice. C. Plasma triglyceride, cholesterol, high density lipoprotein and low density lipoprotein levels of E-/- *ApoE*-/- and E-/- *ApoE*-/-+PAM group. D. Plasma triglyceride, cholesterol, high density lipoprotein and low density lipoprotein levels of E-/- *ApoE*-/- ShCON and E-/- *ApoE*-/-+ShKLF4 group.**


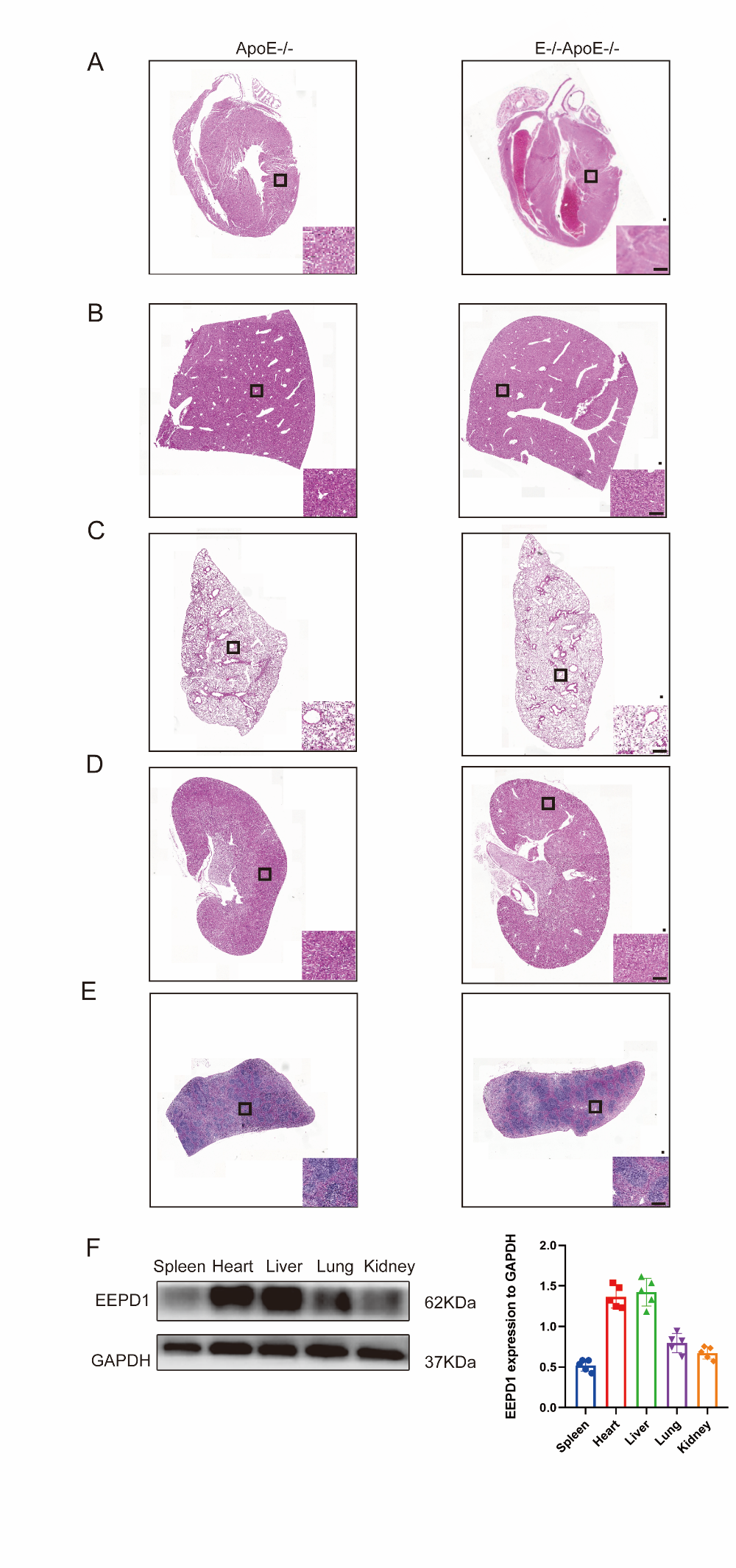


**Supplementary Figure3 A. HE staining of heart tissues from *ApoE*-/- and E-/-*ApoE*-/- mice including one-time magnification images and locally five-times magnified images, with scale bar of 20 μm. B. HE staining of liver from *ApoE*-/- and E-/-*ApoE*-/- mice  including one-time magnification images and locally five-times magnified images, with scale bar of 20 μm. C. HE staining of lung from *ApoE*-/- and E-/-*ApoE*-/- mice  including one-time magnification images and locally five-times magnified images, with scale bar of 20 μm. D. HE staining of kidney from *ApoE*-/- and E-/-*ApoE*-/- mice  including one-time magnification images and locally five-times magnified images, with scale bar of 20 μm. E. HE staining of spleen from *ApoE*-/- and E-/-*ApoE*-/- mice  including one-time magnification images and locally five-times magnified images, with scale bar of 20 μm. F. Representative immunoblots** **showing EEPD1 protein level in spleen, heart, liver, lung and kidney.**


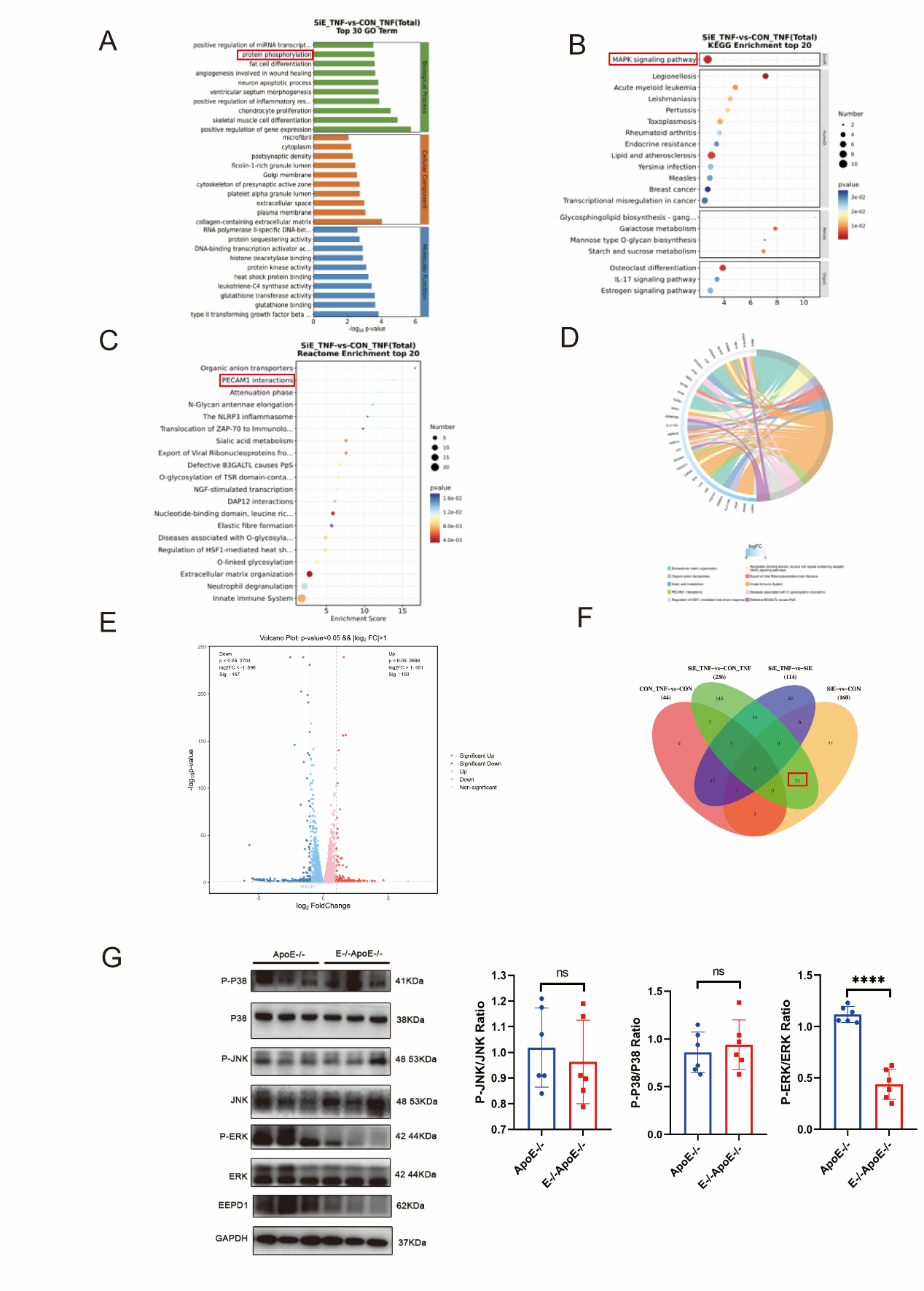


**Supplementary Figure4 A. Barplot of top 30 enriched GO terms between Si*EEPD1*+TNFα and SiCon+TNFα. GO = gene ontology. B. Bubbleplot of the top 20 KEGG enrichment terms between Si*EEPD1*+TNFα and SiCon+TNFα. C. Bubbleplot of the top 20 Reactome enrichment terms between Si*EEPD1*+TNFα and SiCon+TNFα. D. Chord plot of the top Reactome enrichment terms between Si*EEPD1*+TNFα and SiCon+TNFα. E. Volcano plot of differential gene between Si*EEPD1*+TNFα and SiCon+TNFα.** **F. The Venn diagram indicates no change in MAPK1 levels in group CON TNF**α **vs CON and group SiE TNF**α **vs SiE, whereas alterations are observed in group SiE TNF**α **vs CON TNF**α **and group SiE vs CON. G. Western blot results indicate a significant increase in ERK phosphorylation levels following EEPD1 knockout, with no significant differences observed in the phosphorylation of P38 and JNK.**


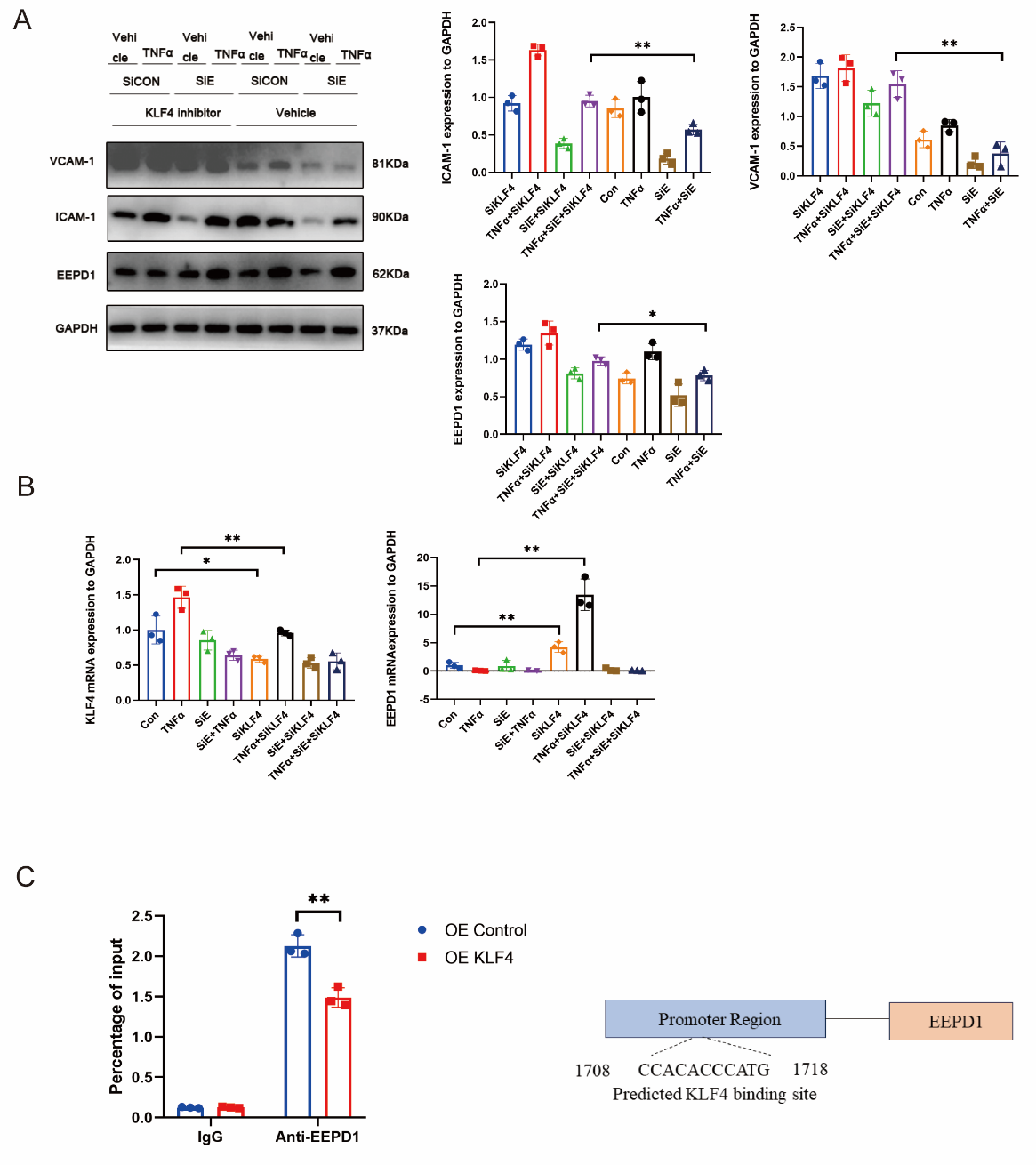


**Supplementary Figure5 A. Western blotting quantification of VCAM-1, ICAM-1, *EEPD1*. B. qPCR quantification of *KLF4* and *EEPD1* mRNA. C. Result of ChIP-qpcr.**
